# Colonization and Maize Growth Promotion Induced by Phosphate Solubilizing Bacterial Isolates

**DOI:** 10.3390/ijms18071253

**Published:** 2017-06-29

**Authors:** Yongbin Li, Xiaomeng Liu, Tianyi Hao, Sanfeng Chen

**Affiliations:** State Key Laboratory of Agrobiotechnology and College of Biological Sciences, China Agricultural University, Beijing 100094, China; ybli@cau.edu.cn (Y.L.); wsw_lxm2012@163.com (X.L.); tianyi_2016@126.com (T.H.)

**Keywords:** maize growth promotion, phosphate solubilizing bacteria, *Paenibacillus*, green fluorescent protein

## Abstract

Phosphorus (P) limits the production of maize, one of the major food crops in China. Phosphate-solubilizing bacteria (PSB) have the capacity to solubilize phosphate complexes into plant absorbable and utilizable forms by the process of acidification, chelation, and exchange reactions. In this study, six bacteria, including one *Paenibacillus* sp. B1 strain, four *Pseudomonas* sp. strains (B10, B14, SX1, and SX2) and one *Sphingobium* sp. SX14 strain, were those isolated from the maize rhizosphere and identified based on their *16S rRNA* sequences. All strains could solubilize inorganic P (Ca_3_(PO_4_)_2_, FePO_4_ and AlPO_4_), and only B1 and B10 organic P (lecithin). All strains, except of SX1, produced IAA, and SX14 and B1 showed the highest level. B1 incited the highest increase in root length and the second increase in shoot and total dry weight, shoot length, and total P and nitrogen (N), along with increased root length. In addition, by confocal laser scanning microscopy (CLSM), we found that green fluorescent protein (GFP)-labeled B1 mainly colonized root surfaces and in epidermal and cortical tissue. Importantly, B1 can survive through forming spores under adverse conditions and prolong quality guarantee period of bio-fertilizer. Therefore, it can act as a good substitute for bio-fertilizer to promote agricultural sustainability.

## 1. Introduction

Maize (*Zea mays* L.), a major food and energy crop, is the third most important food crop in China after wheat and rice [1]. Phosphorus (P) functions as the second largest nutrient after nitrogen, is essential for plant growth and development [2], and takes part in some major metabolic processes, such as signal transduction, macromolecular biosynthesis, energy transfer, photosynthesis, respiration and macromolecular biosynthesis [3]. However, P deficiency constrains biomass accumulation in the agroecosystem [4]. Although there are large amounts of inorganic and organic forms in soils, only 0.1% total P is available for assimilation by plants [5]. This leads to strong demand for the application of P fertilizer. However, frequent application of P fertilizer is not only expensive but also eco-unfriendly [6,7]. It may lead to algal blooms by causing the eutrophication of lakes [8] and the loss of soil fertility [9]. In fact, the P fertilizer utilization is less than 30% [10], because soluble P is quickly fixed by reacting with free Al^3+^, Ca^2+^, Mg^2+^, and Fe^3+^ upon its application in the soil [10,11]. Leaching and run-off also result in a loss of P fertilizer [12]. P ore, a finite resource, is the primary original material of P fertilizer. Cordell et al. [13] estimated these high-quality P ores may run out within 50–100 years. Khan et al. [14] indicated mobilization of all insoluble P accumulated in agricultural soils would be enough to be utilized by crops for about 100 years. Therefore, applying bio-fertilizers possessing P-solubilizing traits shows promise as an alternative to chemical fertilizer [10].

Most plant-growth-promoting rhizobacteria (PGPR) can promote plant growth by multiple mechanisms, such as nitrogen fixation, phosphate solubilization, indole-3-acetic acid (IAA) production and siderophore secretion, which may contribute to differential growth responses of inoculated plants [15,16]. Phosphate solubilizing bacteria (PSB) play a critical role in biogeochemical cycling models of soluble and insoluble P cycling forms in agricultural ecosystems. They can convert insoluble P (inorganic and organic forms) to soluble P (ionic phosphate and low molecular-weight organic phosphate) accessible to plants [17,18,19]. The main mechanisms contain: (a) dissolving directly mineral complexes to release soluble P [20] and (b) chelating Ca^2+^, Fe^3+^, and Al^3+^ to form stable complexes available for plants [21,22]. Insoluble organic P can be converted into available forms for plant uptake through mineralization by PSB [23], and mineralization of most insoluble organic P is performed mainly by phosphatase enzymes such as acid phosphatases [20]. PSB can secrete low molecular weight organic acids such as gluconic, acetic, fumaric, and citric acid to solubilize inorganic P complexes [24,25].

PSB of *Pseudomonas*, *Acinetobacter*, *Sinorhizobium*, and *Arthrobacter* could promote growth of pea, cucumber, medicago truncatula, and rice, respectively [26,27,28,29,30]. For instance, Yazdani et al. [30] demonstrated application of PSB could reduce the application rate of P fertilizer by 50% without reducing maize yield in field trials. The endophytic strains *Pseudomonas fluorescens* L321 isolated from *Miscanthus giganteus* improved pea growth promotion [26]. Moreover, PSB might also enhance drought tolerance in plant [31] and promote phytoremediation of contaminated soil by heavy metal [32,33].

The green fluorescent protein (GFP) from the jellyfish *Aequorea victoria* is a small protein [34]. It has been widely applied in protein localization and gene expression in various organisms. Valdivia et al. [35] explored the potential GFP applications of host-parasite interactions, indicating GFP expression in the pathogens did not reduce bacteria survival, their infective ability to mammalian cells or their survival within macrophages. Now, GFP has been successfully employed to observe plant-PGPR interaction [36,37,38]. However, until now, no data have been available with respect to the colonization of PSB.

We have isolated bacteria from main maize growing areas of North China. These areas share about a third of total maize planting area of China [39]. It was hypothesized the plant rhizosphere of these areas have some potential fine PSB that can greatly promote plant P-nutrition and growth, especially for maize. With this purpose, six PSB strains were obtained and a comparative research was carried out for phosphate solubilization, IAA production and maize growth promotion. In addition, the colonization pattern and ability of *Paenibacillus* sp. B1 in plant tissues were addressed using GFP-label combined with confocal laser scanning microscopy (CLSM). To the best of our knowledge, this is the first report of *Paenibacillus* isolated from maize rhizosphere that presents a phosphate solubilization ability.

## 2. Results

### 2.1. Characterization and Identification of Isolated Phosphate-Solubilizing Bacteria

**Figure 1:**
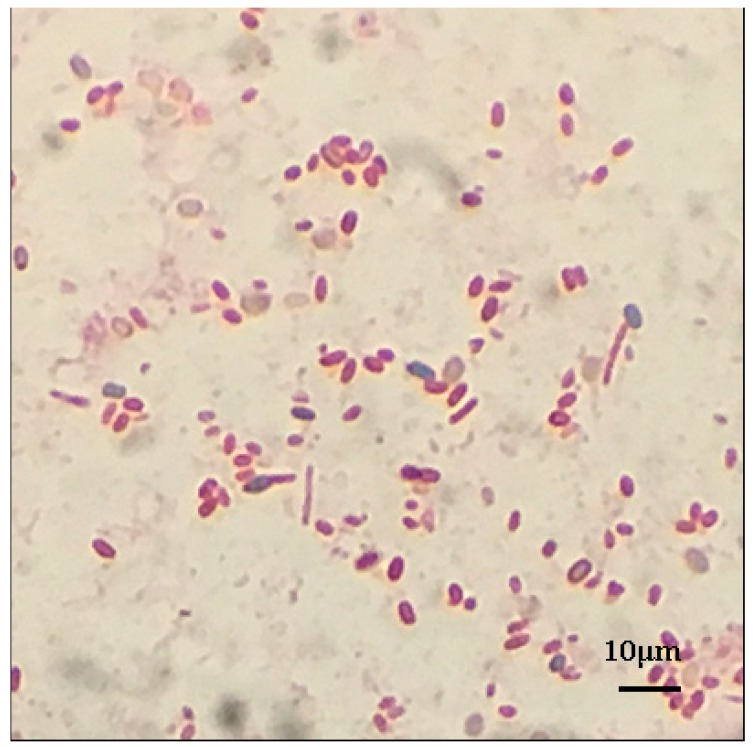
Spore stain of *Paenibacillus* sp. B1. Red represents the cells, and blue the spores.

Six bacteria strains producing obvious transparent zone in Pikovskaya (PVK) medium were purified from rhizosphere soil (Table 1). Strains B1, B10, and B14, which were obtained from Beijing, China, showed white colony with rods. Strains SX1, SX2, and SX14, which were obtained from Shanxi Province, China, showed milky white colony with short rods, white colony with short rods and vitelline colony with rods, respectively. All strains except B1 were Gram-negative, and B1 was the only endospore-forming bacteria (Figure 1).

Based on the analysis of the *16S rRNA* gene sequence, B1 was presumably identified as *Paenibacillus* sp., SX14 as *Sphingobium* sp. and others as *Pseudomonas* sp., all except SX14 showing 99% homologies with the respective sequences in the GenBank database (Table 1). The *16S rRNA* gene sequences were submitted to the GenBank nucleotide database under the accession numbers KY111475, KY111476, KY111477, KY122023, KY122024, and KY122025.

**Figure 2:**
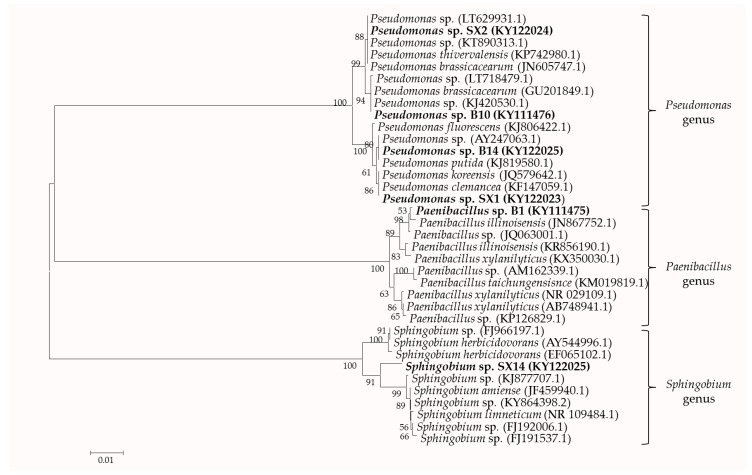
Phylogenetic tree based on *16S rRNA* sequence shows the position of isolated strains with other closely related strains. These *16S rRNA* sequences of related strains were downloaded from NCBI GenBank database. The tree was structured using neighbor joining method, with the bootstrap analyses of 1000 cycles. Only bootstrap values greater than 50% is shown at the branching points. Bar represents sequence divergence of 0.01 nucleotides. Strains isolated in this study are underlined with the bold letters.

Phylogenetic analysis (Figure 2) based on the neighbor joining method revealed three different genera involved *Pseudomonas*, *Paenibacillus*, and *Sphingomonas*, with high bootstrap values. Strains B10, B14, SX1, and SX2 were affiliated *Pseudomonas* cluster, while B1 and SX14 belong to *Paenibacillus* and *Sphingomonas*, respectively.

### 2.2. Qualitative and Quantitative Analysis of Phosphate Solubilization Ability of Isolated Phosphate-Solubilizing Bacteria

After inoculation of these strains on modified PVK medium plates for 7 days at 30 °C, the strains generated visible halo zones, and showed considerable variation in phosphate solubilization index (PSI) ranged from 2.03 to 7.64 (Table 2), and B14 showed the largest PSI, reaching up to 7.64.

All strains solubilized Ca_3_(PO_4_)_2_, AlPO_4_, and FePO_4_ accompanied with a decrease of the pH of supernatants in the National Botanical Research Institute’s Phosphate (NBRIP). Only B1 and B10 solubilized lecithin (Table 3). The concentration of soluble P released from Ca_3_(PO_4_)_2_ ranged from 71.7 to 530 mg·L^−1^ and the strains SX2 secreted the highest amounts of soluble P (530 mg·L^−1^). The values of soluble P released from AlPO_4_ ranged from 9.53 to 95.18 mg·L^−1^ and the strains B1 secreted the highest amounts of soluble P into the medium (95.2 mg·L^−1^) and showed the lowest value of pH in their supernatants (3.13). The values of soluble P released from FePO_4_ ranged from 4.59 to 47.2 mg·L^−1^ and the strains B1 and SX2 secreted the highest amounts of soluble P in to the medium (23.0 and 47.2 mg·L^−1^, respectively). Only both strains B10 and B1 could solubilize organic lecithin in the NBRIP broth medium (2.04 and 0.43 mg·L^−1^, respectively).

Correlation analysis indicated that the values of PSI and the concentration of soluble P in liquid medium NBRIP medium containing Ca_3_(PO_4_)_2_ had a low positive relationship (*r* = 0.526). Between soluble P released in liquid medium NBRIP with Ca_3_(PO_4_)_2_ and pH of supernatants having a low negative relationship (*r* = −0.524), the values of soluble P released from AlPO_4_ and FePO_4_ and supernatants pH was not directly correlated (*r* = 0.001 and *r* = 0.235, respectively). With the exception of control, the pH of all the bacterial supernatants was below 6.0, because PSB can excrete organic acids into the medium, i.e., gluconic acid and citric acid, which can reduce the pH of medium and solubilize mineral phosphates.

All strains, with the exception of SX1, produced IAA. SX14 and B1 produced the highest amount of IAA (22.7 and 20.3 mg·L^−1^, respectively).

### 2.3. Growth Promotion Potential of Isolated Phosphate-Solubilizing Bacteria

**Figure 3:**
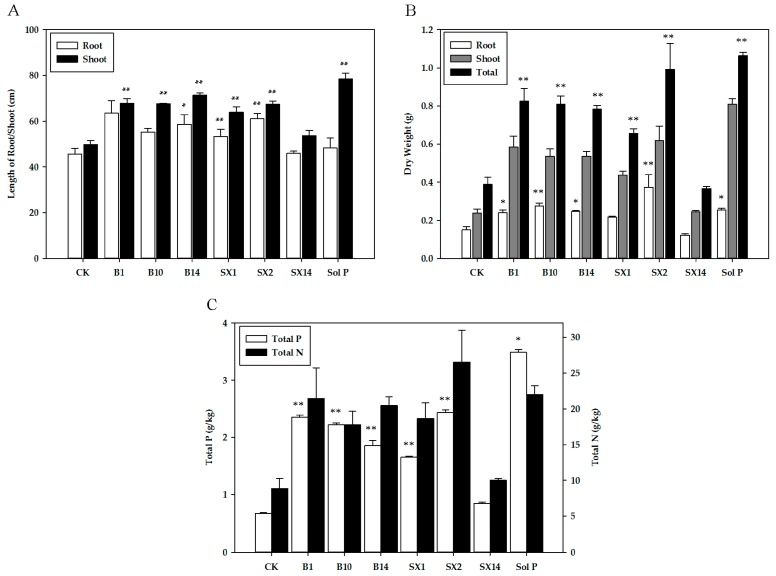
Effects of phosphate solubilizing bacteria inoculation on shoot and root length (**A**), dry weight (**B**), and total P and N of plant (**C**). The values are means from three biological replicates. The bars represent the standard errors of the means. CK: un-inoculated seedlings. Sol P: un-inoculated seedlings watered with nutrient solution containing 0.25 mM KH_2_PO_4_. Single asterisks or double asterisks (* or **) indicate significant differences between negative control and other treatment determined by LSD at *p* < 0.05 or *p* < 0.01.

Overall, inoculation with SX2 and B1 exerted substantial increase in plant growth parameters. Most isolated PSB strains greatly increased the length of root and shoot (Figure 3A), and plants inoculated with B1 showed maximum increase in root length (39.2%), followed by those inoculated with SX2 (33.5%) and B14 (28.4%). The ones inoculated with B14 showed a maximum increase in the shoot length (43.1%), followed by B1 (35.9%) and B10 (35.5%). In Figure 3B, inoculation with SX2 significantly outperformed all six of the other PSB inoculated treatments for root dry weight, shoot dry weight, and total dry weight, and inoculation with B1 had the second highest increase of shoot dry weight and total dry weight. Inoculation with PSB strains significantly enhanced P and N uptake (Figure 3C). The seeding inoculated with SX2 had the highest total N (26.5 mg·L^−1^) and total P (2.44 mg·L^−1^) among six treatments. And the seedling with B1 ranked second. Especially total N (21.5 mg·L^−1^), which was even equal to the one in Sol P (22 mg·L^−1^). In addition, B10, B14, and SX1 also had significantly positive effects on the P and N uptake. Compared with CK, inoculation with SX14 had no obvious effect on N uptake, but also promote P uptake. As expected, un-inoculated plants treated with the soluble P (positive control) yielded the maximum quantity of biomass (shoot length, shoot dry weight, total weight dry, and total P).

### 2.4. Fluorescent Microscopy of GFP-Labelled Paenibacillus sp. B1 in Maize Root

**Figure 4:**
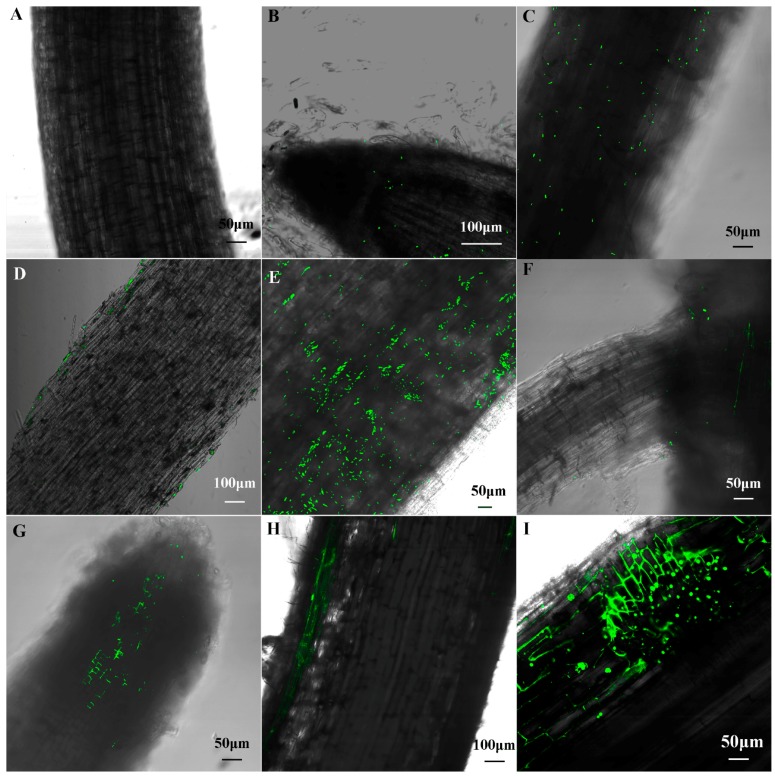
Colonization of the GFP-labeled cells in maize root. (**A**) Non-inoculated control plant; (**B**–**D**) Colonization patterns of the GFP-labeled *Paenibacillus* sp. B1 in maize root after one day of inoculation; (**E**–**G**) Colonization patterns after three days of inoculation; (**H**,**I**) Colonization patterns after five days of inoculation. Green dot represents the GFP-labeled *Paenibacillus* sp. B1.

No bacterial cells were observed in the un-inoculated control maize seedlings (Figure 4A). One day after inoculation, a small number of cells with green fluorescence were found to colonize on the surface of root tip and primary root (Figure 4B,C). Longitudinal sections of primary root showed these cells only colonized on the surface of root (Figure 4D). Three days after inoculation, more cells were observed on the surface of root (Figure 4E), and, some bacteria were observed in the junction of the primary and lateral root (Figure 4F) and longitudinal sections of root showed some cells had distributed to the cell gap of the meristem region (Figure 4G), indicating that B1 strains invaded into inside the meristem region of maize root firstly. After seedlings were infected five days, the bacteria cells were found within epidermal cells and the lateral root primordial (Figure 4H,I). Seven and nine days after inoculation, B1 was not further observed within the cortex and vascular bundle (figure not present).

## 3. Discussion

The narrow zone, which is particularly affected by the root activity, is designated as the rhizosphere [40], which is a habitat of a unique population of plant-associated microorganisms-beneficial, harmful, or neutral. Those beneficial rhizobacteria could successfully colonize plant roots and positively enhance plant growth through various properties, e.g., nitrogen fixation, IAA production, phosphate solubilization, siderophore secretion, or induced systemic resistance (ISR) [41,42]. Lower effective P in soil and large available P demanded for plant growth is contradictory, so the ability of phosphate solubilization is an important parameter for assessing PGPR [43]. North China is the prime area for maize production in China and main crop rotations is winter wheat-summer maize [44]. In these areas, the farmers apply P fertilizer about 75 kg·hm^−2^ for maize production [45]. To overcome dependency on P fertilizer and reduce the cost of production in agriculture, we isolated six strains from the maize rhizosphere. Phylogenetic analysis showed three clusters including *Pseudomonas*, *Paenibacillus*, and *Sphingomonas*. The cluster of *Pseudomonas* encompasses two subgroups. In fact, *Pseudomonas* sp. SX2 clustered with the *Pseudomonas* sp. B10 and *Pseudomonas* sp. B14 with *Pseudomonas* sp. SX1. In addition, *Paenibacillus* sp. B1 grouped in a separate subcluster with *P. illinoisensis* JN867752.1. Though *Sphingomonas* sp. SX14 grouped in the cluster of *Sphingomonas* with high bootstrap values, it did not group in a separate subcluster with others. Some members included in the phylogenetic tree have been reported because of their plant growth promoting or biocontrol functions. *Pseudomonas brassicacearum* Zy-2-1 (GU201849.1) was isolated from root nodules of the wild legume *Sphaerophysa salsula*, and inoculation with Zy-2-1 had impact on growth-promoting activity, nodule number and average nodule weight of *S. salsula* plants [46]. *Pseudomonas* sp. S50 (KT890313.1), isolated from potato, presented the anti-*Phytophthora* potential through the emissions of volatile organic compounds [47]. *Pseudomonas brassicacearum* J12 (JN605747), isolated from tomato, could serve as an antagonist against *Ralstonia solanacearum* [48].

In this study, six PSB were isolated on PVK agar plate containing insoluble tricalcium phosphate. Nautiyal [49] demonstrated the NBRIP medium presented the same efficiency as PVK medium in a plate assay, however, NBRIP medium could reach three-fold higher than PVK medium in a broth assay. The NBRIP broth assay was considered more reliable than the plate assay in measuring a strain as PSB [50]. In our qualitative and quantitative assays, contradictory results were observed. Of the six strains, B14 produced the largest value of PSI, but the fourth highest soluble P in the liquid NBRIP medium containing Ca_3_(PO_4_)_2_. Similar results were found when measuring P solubilization ability [51]. The value of PSI is not adequate for estimating the P solubilization ability of PSB, although it is simple and fast. Therefore, further quantitative determination this trait in NBRIP broth medium was of great significance. There is no metal-P complex that can act as the general selection factor for PSB because of the variability of soils in pH and some chemical properties [52]. Hence, it is imperative to measure the P solubilization ability using a combination of two or more P complexes together. So insoluble inorganic P (Ca_3_(PO_4_)_2_, AlPO_4_, and FePO_4_) and organic P (lecithin) were elected to quantify this ability of PSB. All strains solubilized Ca_3_(PO_4_)_2_ to a greater extent than other insoluble P. Similar results were reported when quantitative determination of the P solubilization ability [53,54] because tricalcium phosphate was considerably more soluble than iron/aluminum phosphate [10]. Our results indicated there was no obvious correlation between the supernatant pH and soluble P released from mineral P by PSB. Acidification may not be the only mechanism of P solubilization, and chelation is also an important one. Only B1 and B10 presented the ability to solubilize organic P (lecithin). The mechanism of organic P solubilization is mineralization by enzymolysis.

PSB can excrete organic acids into the soil, i.e., gluconic acid and citric acid, which can reduce the pH of soil and solubilize mineral phosphates for the plant growth and development [26]. PSB can also adsorb micronutrients, e.g., iron, zinc, and copper, around the root of crops by secreting organic acids, and then stimulate the rapid growth of plants and enhance plant cold, drought, and salinity tolerance [55]. The P solubilization ability appears frequently associated with PGPR, however, it is not necessarily related with the ability to promote plant growth [54,56,57]. The “real” PSB must be directly dedicated to P plant nutrition [58]. So, IAA production was also analyzed in this study and SX14 and B1 secreted the highest amount of IAA. The pot experiment showed maize seedlings inoculated with PSB, except *Sphingobium* sp. SX14, grew faster than the negative control. Inoculation with B1 and SX2 showed maximum beneficial effects on biomass accumulation, P uptake, and N uptake, which we tentatively link to the abilities to produce high amounts of IAA, as well as soluble P. IAA can positively promote plant growth, such as lateral root development and root elongation [15,16]. It is interesting that inoculation had a significantly positive effect on N uptake. Maize seedlings inoculated with SX2 and B1 accumulated the highest amount total N among all those inoculated. Yang et al. [59] proved the fungal *Phomopsis liquidambari* colonization could induce several genes related to N uptake and N metabolism to express differentially to enhance N utilization efficiency in rice. Therefore, we hypothesize SX2 and B1 colonization can also effectively improve N uptake in maize just as described above. SX14 did not induce the plant-promoting growth, though, owing the abilities of P solubilization and IAA production. Whether SX14 is a kind of beneficial plant-associated microorganism needs to be investigated further.

Of the six PSB strains, *Paenibacillus* sp. B1 and *Pseudomonas* sp. SX2 presented a positive plant growth response and the finest PGPR traits (P solubilization and IAA production). The PGPR can function only if they successfully colonize in the plant roots [60]. GFP has been widely used for studying plant-microbe interactions [37,38,61,62]. *Pseudomonas*, a non-endospore-forming bacterium, could colonize velamen and core parenchyma of *Dendrobium nobile* [63]. *P. polymyxa* WLY78 (azotobacter) could colonize wheat, maize, and cucumber [38], though some PGPR such as *Pseudomonas* used in commercial bio-fertilizers, the utilization of bio-fertilizers are still a small fraction in the agriculture industry. In the first instance, PGPR is hindered by environmental variables, such as pH, salinity, and climatic conditions in the field application [10]. Second, these existing bio-fertilizers have many disadvantages, such as a short retention period and unstable activity, posing great difficulties to practical application. However, the ability to form endospores can allow PGPR to survive in a wide range of environmental variables [64], thus facilitating to produce a high-quality bio-fertilizer having long retention period. Taken together, *Paenibacillus* sp. B1 is considered as a better candidate to produce bio-fertilizer than *Pseudomonas* sp. SX2. Hence, only B1 was chosen to employ a colonization assay in the gnotobiotic model system, and it has been demonstrated the gnotobiotic model system could reflect the natural colonization pattern in soil [65]. GFP-labelled B1 could successfully colonize on the surface and within the epidermis cells of maize roots.

## 4. Materials and Methods

### 4.1. Soil Sampling and Phosphate-Solubilizing Bacteria Isolation

Rhizosphere soil samples were derived from two different sites, including Wenshui County, Shanxi Province (112.03° E and 37.43° N) and Beijing City (116.10° E and 40.08° N), China. The plant was uprooted by scoop and loosely adhering soil was removed by shaking gently, then the tightly-adhering soil around the root, which is regarded as rhizosphere soil, was placed in a sterilized valve bag and transported to the laboratory for further isolation of PSB strains as soon as possible.

PSB strains were isolated by the standard dilution plating technique on modified PVK medium, which was made as follows: C_6_H_12_O_6_, 10.0 g; Ca_3_(PO_4_)_2_, 5.0 g; (NH_4_)_2_SO_4_, 0.5 g; NaCl, 0.3 g; MgSO_4_·7H_2_O, 0.3 g; KCl, 0.3 g; MnSO_4_·4H_2_O, 0.03 g; FeSO_4_·7H_2_O, 0.03 g; agar, 18.0 g, H_2_O 1 L; pH (at 25 °C) 7.2 ± 0.2 [66]. The plates were incubated for seven days at 30 °C, and then colonies with transparent zones were picked and purified three times on Luria Bertani (LB) agar plates and then stored at 4 °C. For long-term storage, these purified PSB strains were stored in the medium with 30% glycerol (*v*/*v*) at −80 °C.

### 4.2. Morphological Characterization of Isolated Phosphate-Solubilizing Bacteria

The bacterial strains were streaked on LB agar plates, followed by incubation of these plates for 48 h at 30 °C, and then colony properties of strains were observed. Cell morphology was described by light microscopy (Olympus CX22LED, Olympus Corporation, Tokyo, Japan). All strains were stained with Gram stain as described by Vincent [67]. Spore staining was performed by Schaeffer-Fulton’s method [68].

### 4.3. Amplication and Sequencing of 16S rRNA Gene and Phylogenetic Analysis

Strains were individually grown on LB broth medium until the OD_600nm_ = 0.8 and then cells were collected by centrifugation. Genomic DNA was extracted and purified using the TIANamp Bacteria DNA Kit (Tiangen Biotech (Beijing) Co., Ltd., Beijing, China) according to the manufacturer’s instructions. The amplication of *16S rRNA* gene sequence was performed with the universal primers 27F (5′ AGAGTTTGATC (AC) TGGCTCAG 3′) and 1492R (5′ CGG (CT) TACCTTGTTACGACTT 3′) as described by Khan et al. [69]. The PCR process was performed according to Shahid et al. [70]. Then *16S rRNA* was sequenced commercially by Shanghai Majorbio Bio-Pharm Technology Co., Ltd., Shanghai, China. The sequenced products were conducted by BLAST software from NCBI (https://blast.ncbi.nlm.nih.gov/Blast.cgi) against the GenBank database.

The phylogenetic tree, which contains our sequencing *16S rRNA* gene and sequences with high similarity scores from the GenBank database, was constructed with MEGA software package(MEGA 6.0, Kumar, Stecher, and Tamura 2015) [71] using the neighbor-joining method. Bootstrap analysis were performed with 1000 cycles, and only bootstrap values greater than 50% were shown at the branch points.

### 4.4. Qualitative and Quantitative Determination of Phosphate Solubilization Ability of Isolated Phosphate Solubilizing Bacteria

A preliminary determination of phosphate-dissolving ability was evaluated by modified PVK agar plate assay. Each strain was prick inoculated in the center of the modified PVK medium containing tricalcium phosphate (Ca_3_(PO_4_)_2_) as insoluble phosphate source with sterile toothpick. All of these plates were incubated at 30 °C for seven days. Experiments were carried in triplicate. The diameter of the colony (d) and halo zone (D) around the colony were measured and PSI was calculated using Equation (1) [72]:

*PSI* = (*D* + *d*)/*d* (1)

In order to quantify the ability of PSB to solubilize inorganic and organic mineralize P complexes, we chose modified NBRIP [49] broth medium, which was made as follows: C_6_H_12_O_6_, 10 g; MgCl_2_, 5 g; MgSO_4_·7H_2_O, 0.25 g; (NH_4_)_2_SO_4_, 0.1 g; KCl, 0.2 g; Ca_3_(PO_4_)_2_, 5 g; H_2_O, 1 L; pH (at 25 °C) 6.75 ± 0.25. FePO_4_ (1 g·L^−1^), AlPO_4_ (2 g·L^−1^), or lecithin (2 g·L^−1^) was used in replacement of Ca_3_(PO_4_)_2_. To replace the calcium source CaCl_2_ (1 g·L^−1^) was added. The PSB strains were grown in LB broth medium at 30 °C for 36 h, respectively, and OD_600nm_ adjusted to 1.0. Then strains were inoculated in 50 mL Erlenmeyer flasks containing 20 mL NBRIP broth medium and incubated at 180 rpm for seven days at 30 °C. The medium was inoculated with *E. coli* JM109 and non-inoculated as the control. Each treatment had three biological replicates. Clear supernatant was obtained by centrifuging at 12,000 rpm for 10 min. And then the concentration of soluble phosphate was analyzed based on the Fiske and Subbarow method [73]. Finally, a subsample was used to record the final pH by pH acidity meter.

### 4.5. Determination of Indole-3-Acetic Acid (IAA) Production

The IAA production was measured by colorimetric analysis. The PSB strains were grown in 50 mL Erlenmeyer flasks containing 20 mL King B broth medium with 0.1% (*w*/*v*) Trp for three days at 30 °C. This medium comprised: peptone, 20 g; K_2_HPO_4,_ 1.15 g; MgSO_4_·7H_2_O, 1.5 g; glycerol, 10 g; H_2_O, 1 L; pH (at 25 °C) 7.2 ± 0.2. Non-inoculated King B broth medium was considered as the negative control. Each treatment had three biological replicates. Clear supernatant was obtained by centrifuging at 12,000 rpm for 10 min. The IAA concentration was measured as described by Glickmann et al. [74]. Briefly, 2 mL (S2/1 method) of Salkowski reagent (1 mL 0.5 M FeCl_3_, 30 mL concentrated H_2_SO_4_, and 50 mL distilled H_2_O) was added to 1 mL of above supernatant. Then, the mixtures were reacted in the dark at room temperature for 30 min. Thereafter, the optical density of reaction solutions at 530 nm was recorded using a spectrophotometer (Shimadzu UVmini-1240, Shimadzu Instruments Suzhou Limited, Soochow, China), and the IAA concentration was estimated against the prepared IAA standard curve.

### 4.6. Growth Promotion Potential of Isolated Phosphate Solubilizing Bacteria

A pot experiment was employed in the greenhouse of the China Agricultural University, Beijing, China to evaluate the effects of these strains on maize growth and development.

The above strains were cultured in 100 mL of LB broth medium for 36 h at 30 °C, respectively, and then cells were harvested by centrifugation at 6000 rpm for 5 min at 4 °C and adjusted to 10^8^ cells mL^−1^ with sterile deionized water. Vermiculite was used as substrate for plant growth in this study, washed three times with deionized water to remove the dissoluble phosphorus, and air-dried at room temperature. 700 g of the air-dried vermiculite was put into each pot (25 cm diameter, 35 cm high). At the same time, Ca_3_(PO_4_)_2_ (3.5 g per pot) was completely blended with the vermiculite as an insoluble phosphate source.

Plump maize seeds were sterilized with 10% sodium hypochlorite for 10 min followed by washing with sterilized deionized water three times and then germinated on sterile Petri dishes containing moist filters in darkness at room temperature for 3–5 days. Homogenous seedlings were selected, some of which were soaked with above bacterial strains suspensions (10^8^ cells·mL^−1^) for 30 min. Then, three seedlings were sown in each plastic pot, and grown in the greenhouse (16 h day/8 h night and 20/8 °C day/night temperature). Every treatment had three pots. The experimental treatments contained: (1) inoculated maize seedlings; (2) un-inoculated maize seedlings as a negative control; and (3) un-inoculated seedlings which were watered regularly with nutrient solution as a positive control containing soluble phosphate (0.25 mM KH_2_PO_4_). Plants were irrigated with nutrient solution (0.65 mM MgSO_4_, 2 mM NH_4_NO_3_, 2 mM CaCl_2_, 0.75 mM K_2_SO_4_, 0.1 mM KCl, 0.25 mM KH_2_PO_4_, 0.2 mM Fe-EDTA, 1 × 10^−3^ mM MnSO_4_, 1 × 10^−3^ mM ZnSO_4_, 1 × 10^−4^ mM CuSO_4_, and 5 × 10^−6^ mM (NH_4_)_6_Mo_7_O_24_, 1 × 10^−3^ mM H_3_BO_3_) in the presence or absence of KH_2_PO_4_, where applicable (30 mL per pot).

When maize seedlings were grown in pots for three weeks, whole plants were harvested and root samples were carefully washed with deionized water to remove the adhering soil. Then three plants were picked out from each treatment (one pot with one plant) to immediately measure the length of the shoot and root. Subsequently, the above plant samples were oven-dried at 105 °C for 30 min to de-enzyme, and then dried at 65 °C until a constant weight for dry weight analysis.

The oven-dried samples were weighed and ground into fine powders. Appropriate amounts of ground tissue were digested by an H_2_SO_4_-H_2_O_2_ mixture at 370 °C and then N concentration was analyzed using a modified Kjeldahl method [75] and P concentration was determined using the standard vanadomolybdate method [76].

### 4.7. Colonization of Maize by GFP-Labelled B1

The transformation vector plasmid pGFP300 [38], which was a shuttle vector in *E. coli* and, expresses GFP from its promoter. This plasmid also contains the tetracycline resistance gene *tet*, which is used as a selectable marker when transformed into *E. coli* and *B. subtilis* cells. A electrotransformation method for constructing GFP-labelled *Panebacillus* sp. B1 was used according to Zhang et al. [77].

The maize seedlings inoculated with GFP-labelled *Panebacillus* sp. B1 was obtained as described above. Then one seedling was sown in each sterile flask (6 cm diameter, 10 cm high) containing 100 mL 1/2 × Murashige and Skoog (MS) semisolid agar medium [78] under gnotobiotic condition and grown in the light growth chamber (27 °C, 70% humidity, and 16 h day/8 h night conditions).

The maize seedling tissues were sampled after sowing for certain times and processed for microscopy (Olympus FluoView™ FV1000 confocal microscope, Olympus Corporation, Tokyo, Japan). These images were collected using FV10-ASW software (Version 03.01.02.02, Olympus Europa Holding GmbH, Hamburg Germany) and assembled using Adobe Photoshop CC 2015 and Adobe Illustrator CS6 (Adobe, San Jose, CA, USA).

### 4.8. Statistical Analysis

Each experimental treatment had three replicates. The results of the measurements were analyzed by analysis of one-way analysis of variance (ANOVA) using SPSS software version 20 (SPSS Inc., Chicago, IL, USA). Means of different treatments were compared using the least significant difference (LSD) at a 0.05 or 0.01 level of probability. The Pearson correlation coefficient was analyzed using double-variable analysis.

## 5. Conclusions

Among all the isolated PSB associated with maize rhizosphere, *Paenibacillus* sp. B1 can produce higher levels of IAA to promote plant growth and development. Secondly, B1 can solubilize various insoluble inorganic P and organic P (lecithin). It can also colonize on maize root surfaces and in epidermal and cortical tissues. More importantly, it can generate spores under unfavorable conditions. Therefore, this strain may be an ideal candidate for producing bio-fertilizer having a long quality guarantee period.

## Figures and Tables

**Table 1:** Characterization and identification of all strains.

Physiological Characteristics				Analysis of *16s rRNA* Gene Sequence		
Strain Code	Location	Cell Morphology	Colony Morphology	Gram Stain	Maximum Similarity of *16S rRNA* with (%)	Accession Number
B1	BJ	Rods	White, regular	+	*Paenibacillus illinoisensis*	KY111475
					NR_113828 (99%)	
B10	BJ	Short rods	White, irregular	–	*Pseudomonas thivervalensis*	KY111476
					KJ420530 (99%)	
B14	BJ	Short rods	white	–	*Pseudomonas fluorescens*	KY111477
					KU977136 (99%)	
SX1	SX	Short rods	milky white	–	*Pseudomonas koreensis*	KY122023
					GQ368179 (99%)	
SX2	SX	Short rods	white	–	*Pseudomonas syringae*	KY122024
					KU977139 (99%)	
SX14	SX	rods	vitelline	–	*Sphingobium mellinum*	KY122025
					NR_133859 (98%)	

BJ, Beijing; SX, Shanxi; +, positive; –, negative; Accession number: the accession number of the strains obtained from the GenBank (NCBI).

**Table 2:** Qualitative analysis of phosphate solubilization ability and IAA production by isolated phosphate-solubilizing bacteria (PSB).

Strain	Qualitative Analysis			IAA Production (mg L^−1^)
	Diameter of Halo/cm	Diameter of Colony/cm	PSI	
B1	0.56 ± 0.06 ^b^	0.40 ± 0.07 ^a^	2.46 ± 0.24 ^c^	20.30 ± 2.69 ^a^
B10	0.59 ± 0.02 ^b^	0.12 ± 0.01 ^c^	5.78 ± 0.26 ^b^	8.97 ± 1.18 ^b,c^
B14	0.95 ± 0.07 ^a^	0.15 ± 0.04 ^c^	7.64 ± 0.62 ^a^	5.84 ± 1.64 ^c^
SX1	0.65 ± 0.08 ^b^	0.14 ± 0.04 ^c^	5.87 ± 0.49 ^b^	ND
SX2	0.61 ± 0.02 ^b^	0.10 ± 0.01 ^c^	7.32 ± 0.35 ^a^	13.20 ± 2.32 ^b^
SX14	0.31 ± 0.02 ^c^	0.30 ± 0.02 ^b^	2.03 ± 0.02^c^	22.70 ± 3.37 ^a^

Data are means ± SE of three independent biological replicates. Bearing different letters in the same row are significantly different from each other according to the least significant difference (LSD) test (*p* < 0.05). ND: not detected.

**Table 3:** Quantitative analysis of phosphate solubilization ability in National Botanical Research Institute’s Phosphate (NBRIP) broth medium with Ca_3_(PO_4_)_2_, AlPO_4_, FePO_4_, and lecithin by isolated PSB.

Strain	NBRIP Medium with Ca_3_(PO_4_)_2_		NBRIP Medium with AlPO_4_		NBRIP Medium with FePO_4_		NBRIP Medium with Lecithin	
	Soluble P (mg·L^−1^)	PH	Soluble P (mg·L^−1^)	PH	Soluble P (mg·L^−1^)	PH	Soluble P (mg·L^−1^)	PH
B1	378.0 ± 35.7 ^b^	5.17 ± 0.03 ^c^	95.2 ± 8.2 ^a^	3.13 ± 0.06 ^d^	23.0 ± 1.9 ^b^	3.52 ± 0.08 ^d^	0.4 ± 0.1 ^b^	4.28 ± 0.02 ^c^
B10	208.9 ± 27.3 ^c^	5.06 ± 0.22 ^c^	9.5 ± 1.3 ^d^	3.51 ± 0.05 ^c^	16.0 ± 2.7 ^b,c^	3.07 ± 0.10 ^e^	2.0 ± 0.2 ^a^	3.58 ± 0.04 ^d^
B14	307.8 ± 13.7 ^b^	5.07 ± 0.41 ^c^	23.4 ± 2.9 ^d^	3.28 ± 0.04 ^d^	4.6 ± 0.4 ^c^	4.12 ± 0.07 ^b^	ND	3.28 ± 0.02 ^e^
SX1	493.1 ± 21.2 ^a^	4.03 ± 0.08 ^d^	56.0 ± 6.4 ^c^	3.59 ± 0.07 ^c^	17.1 ± 2.3 ^b^	3.91 ± 0.04 ^c^	ND	2.93 ± 0.04 ^f^
SX2	529.7 ± 45.9 ^a^	5.49 ± 0.10 ^b,c^	73.0 ± 5.4 ^b^	4.11 ± 0.07 ^b^	47.2 ± 8.4 ^a^	4.09 ± 0.07 ^b^	ND	3.66 ± 0.03 ^d^
SX14	71.7 ± 12.9 ^d^	5.82 ± 0.03 ^b^	16.4 ± 1.4 ^d^	3.62 ± 0.11 ^c^	16.2 ± 2.4^b,c^	3.19 ± 0.08 ^e^	ND	4.75 ± 0.03 ^b^
JM109	ND	6.43 ± 0.03 ^a^	ND	6.48 ± 0.06 ^a^	ND	6.63 ± 0.07 ^a^	ND	6.52 ± 0.18 ^a^

Data are means ± SE of three independent biological replicates. Bearing different letters in the same row are significantly different from each other according to the LSD test (*p* < 0.05). ND: not detected.

## Abbreviations

P	Phosphorus
N	Nitrogen
PSB	Phosphate solubilizing bacteria
CLSM	Confocal laser scanning microscopy
PGPR	Plant-growth-promoting rhizobacteria
PVK	Pikovskaya
PSI	Phosphate solubilization index
NBRIP	National Botanical Research Institute Phosphate
ISR	Induced systemic resistance
LSD	Least significant difference
